# A multidisciplinary management algorithm for brain metastases

**DOI:** 10.1093/noajnl/vdac176

**Published:** 2022-11-30

**Authors:** Alexander Ramos, Alexandra Giantini-Larsen, Susan C Pannullo, Andrew Brandmaier, Jonathan Knisely, Rajiv Magge, Jessica A Wilcox, Anna C Pavlick, Barbara Ma, David Pisapia, Hani Ashamalla, Rohan Ramakrishna

**Affiliations:** Department of Neurological Surgery, Weill Cornell Medicine, New York Presbyterian Hospital, New York, New York, USA; Department of Neurological Surgery, Weill Cornell Medicine, New York Presbyterian Hospital, New York, New York, USA; Department of Neurological Surgery, Weill Cornell Medicine, New York Presbyterian Hospital, New York, New York, USA; Department of Radiation Oncology, Weill Cornell Medicine, New York Presbyterian Hospital, New York, New York, USA; Department of Radiation Oncology, Weill Cornell Medicine, New York Presbyterian Hospital, New York, New York, USA; Department of Neurology, Weill Cornell Medicine, New York Presbyterian Hospital, New York, New York, USA; Department of Neurology, Weill Cornell Medicine, New York Presbyterian Hospital, New York, New York, USA; Department of Neurology, Memorial Sloan Kettering Cancer Center, New York, New York, USA; Department of Oncology, Weill Cornell Medicine, New York Presbyterian, New York, New York, USA; Department of Oncology, Weill Cornell Medicine, New York Presbyterian, New York, New York, USA; Department of Pathology, Weill Cornell Medicine, New York Presbyterian, New York, New York, USA; Department of Neurology, Weill Cornell Medicine, New York Presbyterian Hospital, New York, New York, USA; Department of Neurological Surgery, Weill Cornell Medicine, New York Presbyterian Hospital, New York, New York, USA

**Keywords:** brain metastases, oligometastatic, precision medicine, stereotactic radiosurgery, whole-brain radiation therapy

## Abstract

The incidence of brain metastases continues to present a management issue despite the advent of improved systemic control and overall survival. While the management of oligometastatic disease (ie, 1–4 brain metastases) with surgery and radiation has become fairly straightforward in the era of radiosurgery, the management of patients with multiple metastatic brain lesions can be challenging. Here we review the available evidence and provide a multidisciplinary management algorithm for brain metastases that incorporates the latest advances in surgery, radiation therapy, and systemic therapy while taking into account the latest in precision medicine-guided therapies. In particular, we argue that whole-brain radiation therapy can likely be omitted in most patients as up-front therapy.

The incidence of brain metastases continues to increase with improved systemic disease control leading to improved survival, with estimation in the literature ranging from 9% to 17%.^1^ The most common primary tumors to metastasize to the brain are breast, lung, and melanoma.^2^ Prognosis varies greatly and multiple prognostic scoring systems have been described,^3,4^ with all finding the most prognostic value in baseline performance status and extracranial control of disease.^2^ Current management paradigms focus on achieving local control with a combination of stereotactic radiosurgery (SRS),^5,6^ whole-brain radiation therapy (WBRT),^7–9^ and surgical resection.^10,11^

WBRT was first described in an autopsy series of metastatic breast cancer to the central nervous system (CNS) in the 1930s and in the 1950s for the treatment of symptomatic brain metastases in patients with poorly controlled malignancies and has been a mainstay of treatment since.^12,13^ As its name implies, the radiation dose is delivered to the entire brain. This regional treatment can address symptomatic and asymptomatic macroscopic brain metastases, and microscopic metastatic disease simultaneously, and can transiently reverse neurologic deficits if tumors regress. WBRT is typically prescribed with a dose of 30 Gray (Gy) delivered over 10 sessions. Various trials of different dosing and scheduling showed no significant advantage compared to this standard treatment.^10,14–16^ In comparison to WBRT, SRS delivers a focused dose of radiation to a specific lesion, typically in limited sessions. While an upper limit of 2.5–3 cm tumor size is typically utilized in randomized trials, often larger tumors are treated with radiosurgery. In general, both therapies provide effective local control as monotherapy and distal brain failure can be reduced for selected histologies with the addition of WBRT.^17^

Here we review the evidence for treatment of brain metastases, from solitary brain tumors to patients with 10 or more lesions. Treatment for 1–4 brain metastases has been studied extensively with an emerging consensus among practitioners, although some controversies still exist. In contrast, management approaches for >4 tumors and especially >10 tumors are mostly unguided by randomized trials. Management recommendations often are guided by local practice patterns and extrapolation from existing data. We suggest based on available evidence that WBRT can likely be omitted for the vast majority of patients given the significant side effect burden. Instead, patients with >4 tumors can be managed with observation or SRS to at risk lesions. Of note, while we have organized this manuscript on the basis of number of brain metastases, one can also consider treatment algorithms based on the total sum volume of metastases treated. While the volume metric is useful from the standpoint of understanding toxicity and prognosis,^18,19^ it is our view that the number of metastases still remains useful and user-friendly from a decision-making standpoint.

## Management of Solitary Brain Metastases

A current standard of care treatment for a solitary brain metastasis is surgical resection followed by radiation therapy to the resection bed for enhanced local control.^20,21^ The benefits of surgical resection were shown in the landmark Patchell study,^11^ which demonstrated increased survival for patients with solitary brain metastasis undergoing resection relative to WBRT, which was used for both arms. This was followed by clinical trials demonstrating enhanced local disease control with the addition of WBRT to resection, although there was no effect on overall survival.^8,22^ In a phase III clinical trial looking at the effect of postoperative SRS versus no radiation after complete resection of 1–3 brain metastases, the 12-month freedom from local recurrence rate was 72% in the cohort receiving postoperative SRS versus 43% in the observation group not receiving postoperative radiation.^23^ SRS was also found to be noninferior to WBRT after surgical resection.^24^ Postoperative SRS to the resection cavity is favored over WBRT due to increased cognitive decline^6,25,26^ and worsened quality of life^27^ seen with adjuvant WBRT. For patients with tumors <3 cm that are asymptomatic, SRS provides comparable local control to surgery combined with postoperative radiation.^28,29^ Consensus contouring guidelines for the clinical target volume (CTV) of SRS to the resection cavity after complete resection have been described. The guidelines suggest including the entire surgical tract in planning and contouring, extending CTV 5 to 10 mm along the dura underneath the bone flap, and leaving a margin of less than or equal to 5 mm along to sinus if the tumor contacted the sinus.^30^ For large and inoperable brain metastases, staged or fractionated SRS is a treatment strategy that provides safe and effective local control for individuals who are unable to undergo resection.^31–33^ A single institution study of 289 patients found that multifaction SRS (3 daily fractions totaling 27 Gy) compared to single-fraction SRS was significantly associated with improved local control (91% vs 77% at 1 year) and decreased radiation necrosis rates.^31^

Therefore, for patients with KPS > 70^34^ and tumors 2–3 cm with significant edema causing symptoms, we recommend surgical resection followed by SRS to the resection cavity, consistent with widely accepted guidelines and level 1 evidence.^20,23,35^ It is important to distinguish poor performance status due to intracranial edema and mass effect that are likely to improve with treatment versus poor performance status due to systemic disease burden. Additional benefits of resection include the rapid discontinuation of steroid therapy typically used to mitigate edema-related symptoms in the brain, and obtaining tissue for histological diagnosis when there is diagnostic uncertainty or to guide targeted therapies. A point of controversy is the lack of significantly increased overall survival demonstrated between observation group and those receiving SRS after post-operation resection, raising the possibility of observation only after resection of a dominant metastasis as a treatment alternative.^23^ However, we favor postoperative radiation therapy given the high likelihood of local recurrence for patients treated with surgery alone.^23^

## Management of 2–4 Metastases

The role of surgery in the treatment of 2–4 metastases is less clearly defined compared to a solitary metastasis. Small observational studies^36,37^ suggest that resection and aggressive treatment of multiple metastases can be beneficial, especially in patients with good baseline performance status and well controlled extracranial disease. With advancements in systemic therapies that increase overall survival, control of oligometastatic brain disease is increasingly important. We recommend surgical resection for dominant or symptomatic metastases in patients with 2–4 tumors, followed by postoperative SRS to the resection cavity^6,23,24,27^ and remaining lesions.

The optimal radiation-based treatment of 2–4 metastases has been the subject of multiple large randomized trials over the past decade,^38^ of which have addressed the topic of combining WBRT with SRS for optimal control.^5,6,22,25,39,40^ In a randomized control trial (RCT) evaluating whether SRS can be used without WBRT, 132 patients were assigned to SRS alone vs SRS with WBRT.^5^ While median overall survival was not statistically different in both groups (*P* = .42; 7.5 WBRT + SRS vs 8 months SRS alone), there was a significant difference in the rates of distal progression at 12 months (*P* < .001, 46.8% WBRT + SRS, 76.4% SRS alone). Another RCT compared surgical resection vs SRS of 1–3 brain metastases followed by either observation or WBRT.^22^ Three hundred fifty-nine patients were assigned to the different cohorts (resection alone, SRS alone, resection followed by WBRT, and SRS followed by WBRT). There were 199 patients in the SRS group of which 99 received post-SRS WBRT. The addition of WBRT again was found to reduce the rate of intracranial relapse but had no effect on the duration of functional independence or overall survival.^22^ Patients assigned to the observation arm after SRS could still receive salvage SRS or WBRT if recurrence was detected; this was the case in approximately 30%–40% of patients. Overall, this led the authors to conclude that WBRT could be withheld with frequent monitoring for recurrence.^22^

Further research in this area focused on cognitive function as a primary outcome. Chang et al. randomized patients to receive SRS + WBRT versus WBRT alone and found that the 1-year freedom from recurrence was 27% for SRS alone and 73% for the WBRT + SRS group. This benefit was offset by a significant decline in memory functioning in the patients receiving WBRT. Furthermore, overall median survival was higher for the SRS only group, an effect mostly attributable to the more aggressive use of salvage surgery, and higher use of chemotherapy in patients assigned to SRS alone.^39^ In a more recent study addressing cognitive outcomes,^25^ Brown et al. looked specifically at quality of life and cognitive outcomes at 3 months, after completion of SRS alone versus SRS + WBRT. There was a significant decline in both cognition (defined as >1 SD from baseline cognitive testing) and overall quality of life in the SRS + WBRT arm. Again, there was a higher rate of distant failure in the SRS alone arm but no effect on overall survival. Finally, a study examining the use of postoperative SRS versus WBRT in patients with 1–4 metastases expanded on the general theme; while there was a higher rate of distant failure and recurrence in the SRS arm, overall survival was not affected. However, there was a significant detriment in both quality of life and cognition in the WBRT group.^6^

A meta-analysis of these earlier RCTs^41^ suggested that distant brain relapse rates were not significantly affected with the omission of WBRT in a subset of patients younger than 50. Furthermore, there was a survival benefit to SRS alone in this cohort. The survival benefit to SRS alone in this cohort was hypothesized to be due to the reduction in quality of life due to WBRT without a corresponding positive impact on overall disease control.^41^

Taken together, these studies suggest that especially for patients with an expected survival of greater than 3 to 6 months, WBRT should be omitted due to significant side effects and lack of significant improvement in overall survival. While there is an increase in intracranial progression with SRS alone, this does not translate into a survival disadvantage. We, therefore, recommend for patients with 1–4 metastases that do not meet criteria for surgical resection that treatment should be SRS and close observation with salvage irradiation as needed.

## Management of 5–10 Metastases

The treatment of a larger number of intracranial metastases remains controversial with no randomized trials to guide management.^14,42^ With the advent of SRS and data indicating no substantial survival benefit with the use of WBRT for the treatment of oligometastases, the use of WBRT has been declining. However, there are still approximately 200 000 patients per year receiving WBRT in the United States.^26^ In a large international survey of practitioners with a radiosurgical practice in 2009, 55% considered treating >5 metastases with SRS alone reasonable, compared to just 22% that considered treating >10 reasonable.^43^ Considering the cognitive and quality of life side effects of WBRT,^6,25,27^ it is preferable to treat with SRS alone if possible.

A landmark prospective multi-institutional trial conducted in Japan addressed the efficacy of SRS without WBRT for the treatment of 5–10 metastases compared with 2–4 and found treatment of 5–10 tumors to be non-inferior.^44^ Of the 1194 patients, 455 had 1 tumor, 531 had 2–4 tumors, and 208 had 5–10 tumors. Inclusion criteria were tumors < 3 cm in maximal diameter, cumulative tumor volume of <15 ml, and KPS > 70 or KPS < 70 where intracranial disease was a significant contributor to their performance status. For this study, individuals were included whose KPS was decreased due to intracranial disease with the hope that KPS would improve after treatment. Individuals who received previous radiation or surgery were excluded. The vast majority of patients had lung cancer (76%). There was no significant difference in survival between the 2–4 and the 5–10 tumor groups (median survival 10.8 months) treated with SRS alone. Additionally, among the patients with multiple tumors, there was no difference in use of salvage therapy, radiation adverse events, neurologic deterioration, or neurologic death. Overall, these data suggest that patients with 5–10 tumors can be treated similarly to patients with 2–4 tumors; they have similar overall survival and their ultimate survival appears to be more dependent on their systemic disease control as there was no difference in the rates of neurologic death.

Concerns about the potential broad applicability of this study have included the relatively homogenous Japanese patient population and an over-representation of lung cancer cases. A recent multi-institutional retrospective study in one North American institution found similar favorable evidence for treatment of 5–15 tumors with radiosurgery alone.^45^ This prospective trial enrolled 478 patients; 220 had 1 tumor, 190 had 2–5, and 68 had 5–15. Fifty-six percentage of patients had lung cancer, 15% melanoma, and 12% breast cancer. There was no significant difference in the need for salvage therapy or toxicity rates in the groups with 2–5 or 5–15 metastases. In comparison to the Japanese group study, there was a trend toward decreased median survival in the 5–15 metastasis cohort; however, the authors note that they were not selected for up-front prognostic factors and >65% had progressive extracranial disease at the time of SRS. A more robust multi-institutional North American study retrospectively investigated 989 patients receiving SRS for 1 tumor, 882 for 2–4 tumors, and 212 for 5–15.^46^ For the patients with 5–15 brain metastasis (BM), the cancers represented were lung (41%), melanoma (27%), breast (16%), other (9%), and renal cell carcinoma (7%). The number of brain metastases had no effect on survival. Salvage SRS was used more frequently in patients with 2–4 tumors, while salvage WBRT was used for progression more commonly in the 5–15 metastases group. In both groups the median time to WBRT was 4.5 months. Overall, the use of salvage therapy was no different between the groups. The study was limited in that performance status and cause of death were not recorded.^46^

A major factor for the continued use of WBRT is tumor subtype. For example, in small cell lung cancer (SCLC), WBRT remains the standard of care for limited or even solitary SCLC due to concern for short-interval progression leading to potential decrease in overall survival if WBRT is omitted. The treatment of brain metastases in SCLC varies greatly, ranging from chemotherapy alone to WBRT/hippocampal avoidance WBRT (HA-WBRT) to SRS depending on clinical factors and provider experience.^47^ For symptomatic brain metastases, WBRT was the most common first line treatment, while chemotherapy alone was the most common first line treatment for asymptomatic patients. Recently, there is literature to support that not all patients with SCLC have rapidly progressive CNS disease and may benefit from first line SRS. In a retrospective study of 710 patients, patients with SCLC and brain metastases saw no significant difference in survival when treated with SRS compared with WBRT.^48^ There are currently 2 clinical trials ongoing exploring first line SRS to treat brain metastases from SCLC. The first is “Stereotactic Radiation in Patients with Small Cell Lung Cancer and 1–10 Brain Metastases: A Single Arm, Phase II Trial” currently enrolling participants (NCT03391362) with an estimated primary completion date of June 30th, 2023 and study completion date of June 30th, 2025. Individuals will be treated with SRS within 14 days of planning MRI and dosing will be based on sizing of lesion.^49^ The other is also a Phase II clinical trial “Stereotactic Radiosurgery (SRS) as Definitive Management for a Limited Number of Small Cell Lung Cancer Brain Metastasis” with an estimated primary and study completion date of December 31st, 2024.^50^ Patients with 5 or less brain metastases are eligible to be included in this study.

Taken together, one prospective trial and 2 large retrospective trials have demonstrated that SRS without WBRT is safe and effective for 4–10 brain metastases. Class 1 evidence to guide practice should be available soon; randomized trials are ongoing.^45,46,51,52^ Furthermore, despite a shorter time to distant brain failure in patients with 4–10 tumors versus 2–4 tumors, there is no difference in median survival or use of salvage therapy.^46^ For patients with high performance status and a dominant symptomatic metastasis, it is reasonable to offer surgery for the dominant lesion followed by SRS. For patients with small masses or tumors in surgically inaccessible locations, or patients unable to tolerate surgery, we recommend SRS to all lesions.

## Management of >10 Metastases

Next, we review the evidence for SRS for >10 metastases and suggest that WBRT can be omitted even in this patient population. In the largest study of patients with >10 tumors treated with SRS alone, Yamamoto et al. analyzed the outcomes of 2553 patients undergoing SRS without WBRT in a single institution over a 13-year period (1998–2011).^53^ The patients were stratified into 2 groups: 1–10 metastases and >10 metastases. There was no statistically significant survival difference between the 2 groups. Those with >10 tumors did not have a higher rate of neurologic death or deterioration, local or distal recurrence, or SRS-related complications. Factors affecting the survival of patients with >10 tumors included young age, systemic disease control, and KPS score. Interestingly, 90% of patients in this study cohort died of systemic disease, suggesting that advances in systemic chemotherapy will prolong survival in concert with SRS-based intracranial control. Another retrospective study of 323 patients receiving SRS at a single center were divided into 4 groups according to the number of tumors: 1–5, 6–10, 11–15, and 15+.^54^ The occurrence of new distant lesions outside of the SRS field was significantly higher in the 15+ tumor group, consistent with other studies.^46^ Overall survival after SRS did not vary between the groups. These results are consistent with a previous large cohort analysis which demonstrated that among 1855 patients treated with SRS, only solitary metastasis demonstrated enhanced survival; outcomes were not correlated with number of metastatic tumors greater than 1.^55^

A smaller single-institution retrospective study of 61 patients receiving SRS for >10 metastases (806 tumors treated) demonstrated the safety and efficacy of SRS in this patient population.^56^ Importantly, many of the individuals included had prior WBRT or SRS, suggesting SRS to multiple tumors can be considered as a salvage therapy. Furthermore, prior WBRT was predictive of the development of radiation-related adverse events, but not prior history of SRS. This study found that controlled systemic disease and high KPS predicted enhanced survival and local control was reported in 81% of patients. A similar study of 53 patients treated at a single institution, of whom 42% had received prior WBRT, found no association between survival and the number of tumors.^57^ Similar results were also found by other groups^58,59^ reporting a median survival for patients receiving SRS (range of reported studies is 4–6 months) that is comparable to patients receiving WBRT.

Importantly, the cumulative total dose of radiation in patients treated for >10 tumors most often does not reach the toxicity level to cause necrosis to the brain tissue. Using radiographic leukoencephalopathy as a surrogate for brain toxicity, studies have demonstrated that a cumulative integral dose of >3 Joules (J) was the only factor predictive of the development of white-matter changes, and these changes were less with single-fraction SRS than with WBRT.^60^ The threshold of >3 J was not dependent on the number of lesions treated, but was met when total tumor volume exceeded 25 cc.^61^ Importantly, cognitive outcomes have not been rigorously evaluated in patients receiving SRS for >10 metastases, although this is being actively examined in multiple open phase III trials.^52^

A long-cited purpose for WBRT is palliation of symptoms for cases of poor performance status in patients with a large number of lesions, although a more recent study has called into question the use of WBRT even for palliation of symptoms.^62^ The QUARTZ trial is the only randomized trial to directly address the question of omission of WBRT in the treatment of widely metastatic non-SCLC (NSCLC). In this multi-institutional study, 538 patients with NSCLC were randomized to either short-course WBRT (20 Gy in 5 fractions) with supportive care including dexamethasone or supportive care alone. These patients were determined to be unsuitable for surgical resection or SRS by local neurosurgeons and radiation oncologists, but the exact criteria employed for individual cases is not known. A potential confounder is that patients who were more robust with a better prognosis were preselected for WBRT and entered into the study by their oncologist, which may skew the results of this trial. Because this was a trial of palliative treatment, there were no exclusions made for KPS; 38% of patients had KPS < 70 and 63% had uncontrolled extracranial disease. While 30% had a solitary brain metastasis, the majority had 2 or more tumors. Strikingly, overall median survival was the same in patients receiving WBRT with supportive care versus supportive care alone, with the caveat that there was a survival benefit seen in younger patients with favorable performance status receiving WBRT with supportive care. Importantly, the study examined quality of life metrics and overall survival and did not report the rate of neurologic death.

The ultimate question in addressing the patient with multiple (>10) brain metastases is: Can WBRT be omitted in these patients in favor of selective SRS and observation? As reviewed above, there is a developing body of literature to suggest that SRS can be performed without WBRT for effective intracranial control and symptom palliation. A more definitive answer may be forthcoming; there are currently 2 active prospective phase III trials in the United States directly comparing WBRT to SRS monotherapy for greater than 4 and up to 20 metastases.^52^ NCT01592968 has finished recruiting, with an estimated study completion date in September 2023. NCT03075072 is currently open and enrolling, with an estimated completion date in 2024. Based on the currently available data, it is reasonable to offer surgical resection for dominant or symptomatic lesion in combination with SRS for individuals with >10 metastases and favorable performance status. Palliative WBRT may be omitted in favor of supportive care if the patient is deemed not a candidate for either stereotactic surgery or resection, with exceptions made for patients < 60 years old and with KPS > 70.

## Management of Brain Metastases With Systemic Therapy

Focal therapy with radiation and neurosurgical resection has long been the mainstay of brain metastasis control. However, with recent advances in the identification of molecular mutations and development of targeted and immunologic therapy, select patients may be appropriate to incorporate CNS-penetrant systemic agents based on their primary cancer, genetic and molecular signatures, and burden of CNS disease into the treatment regimen.^63^ The most recent American Society for Clinical Oncology/Society for Neuro-Oncology/American Society for Radiation Oncology guidelines support the use of CNS-penetrant systemic therapy in carefully selected patients.^64^

In the last decade, numerous somatic driver mutations involving genes such as EGFR, ROS1, ALK, and BRAF have become the target of modern drug development.^63^ The resultant explosion of new FDA-approved targeted agents for the treatment of subsets of lung,^65–70^ breast,^71–74^ and melanoma^75^ malignancies has revolutionized the modern landscape of oncologic care (Table 1). Many of these agents are small-molecule tyrosine kinase inhibitors (TKIs), and at adequate dosing demonstrate clinical activity against brain metastases.

**Table 1.** Select Clinical Trials of Targeted Therapy and Immunotherapy for Brain Metastases

Drug	Reference	Phase	Study Arms	*N* With BM	Prior RT^a^	Prior TKI/ICI^b^	Endpoints			
							CNS ORR	CNS DCR	Response Duration^^^	Survival^c^
NSCLC										
EGFR inhibitors										
Osimertinib	Wu et al. (2018)^65^ (AURA-3)	III	Osimertinib 80mg for T790M-mutant	75	37%	100%	40.0%	87.0%	11.7 months^1^	N/A
			Platinum-pemetrexed	41	49%	100%	17.0%	68.0%	5.6 months^1^	N/A
	Reungwetwattana et al. (2018)^66^ (FLAURA)	III	Osimertinib	61	25%	0%	66.0%	90.0%	16.5-NE months^1^	N/A
			Gefitinib or erlotinib	67	24%	0%	43.0%	84.0%	13.9 months^1^	N/A
	Park et al. (2020)^76^	II	Osimertinib 160 mg for T790M- mutant	40	45%	100%	55.0%	77.5%	7.6 months^2^	16.9 months
ALK inhibitors										
Alectinib	Peters et al. (2017)^67^ (ALEX)	III	Alectinib	58	N/A	0%	59.0%	N/A	17.3-NE months^3^	N/A
			Crizotinib	64	N/A	0%	26.0%	N/A	3.7 months^3^	N/A
Brigatinib	Camidge et al. (2018)^68^ (ALTA)	I/II + II	I/II	15	N/A	92%	53.0%	87.0%	14.6 months^1^	N/A
			ALTA Arm A (90 mg)	26	N/A	100%	46.0%	85.0%	15.6 months^1^	N/A
			ALTA Arm B (180 mg)	18	N/A	100%	67.0%	83.0%	18.4 months^1^	N/A
Lorlatinib	Solomon et al. (2018)^69^	II	Lorlatinib	81	N/A	100%	63.0%	N/A	14.5 months^4^	N/A
	Shaw et al. (2020)^70^ (CROWN)	III	Lorlatinib	38	N/A	0%	66.0%	N/A	NE-NE months^3^	N/A
			Crizotinib	40	N/A	0%	20.0%	N/A	9.4 months^3^	N/A
Immunotherapy										
Pembrolizumab	Goldberg et al. (2020)^83^	II	Pembrolizumab							
			PD-L1 > 1%	37	N/A	0%	29.7%	35.1%	2.3 months^1^	11.4 months
			PD-L1 < 1%	5	N/A	0%	0.0%	N/A	N/A	4.8 months
	Gadgeel et al. (2020)^84^ (KEYNOTE-189)	III	Pembrolizumab + Pemetrexed + Carboplatin	73	N/A	0%	N/A	N/A	N/A	19.2 months
			Placebo + Pemetrexed + Carboplatin	35	N/A	0%	N/A	N/A	N/A	7.5 months
Atezolizumab	Gadgeel et al. (2019)^85^ (OAK)	III	Atezolizumab	61	90%	0%	N/A	N/A	NR months^6^	16.0 months
			Docetaxel	62	82%	0%	N/A	N/A	9.5 months^6^	11.9 months
Breast										
HER2 targeting agents										
Lapatinib	Bachelot et al. (2013)^71^ (LANDSCAPE)	II	Lapatinib + Capecitabine	45	0%	0%	57.0%	92.9%	5.5 months^5^	17.0 months
Neratinib	Freedman et al. (2019)^72^	II	Neratinib + Capecitabine							
			Lapatinib-naïve	37	35%*	0%	49.0%	82.0%	5.5 months^2^	13.3 months
			Lapatinib-treated	12	58%*	100%	33.0%	66.0%	3.1 months^2^	15.1 months
Tucatinib	Lin et al. (2020)^73^ (HER2CLIMB)	II	Tucatinib + Capecitabine + Trastuzumab	198	71%	0%	47.3%	N/A	9.9 months^1^	18.1 months
			Placebo + Capecitabine + Trastuzumab	93	69%	0%	20.0%	N/A	4.2 months^1^	12.1 months
Trastuzumab Deruxtecan	Modi et al. (2019)^74^	II	Trastuzumab Deruxtecan for HER2+	24	N/A	N/A	N/A	N/A	18.1 months^2^	N/A
Melanoma										
Immunotherapy										
Ipilimumab + Nivolumab	Tawbi et al. (2021)^82^	II	Ipilimumab + Nivolumab x 4 doses, then Nivolumab monotherapy							
			Asymptomatic	101	N/A	N/A	53.5%	57.4%	54.1% at 36-months^1^	71.9% at 36-months
			Symptomatic	18	N/A	N/A	16.7%	16.7%	18.9% at 36-months^1^	36.6% at 36-months
	Long et al. (2018)^80^	II	Ipilimumab + Nivolumab x 4 doses, then Nivolumab monotherapy for asymptomatic, untreated BM	35	0%	0%	45.7%	N/A	2.9-NR months^1^	8.5-NR months
			Nivolumab monotherapy for asymptomatic, untreated BM	25	0%	0%	20.0%	N/A	2.5 months^1^	18.5 months
			Nivolumab monotherapy for symptomatic BM, recurrent BM after local therapy, or leptomeningeal metastases	16	50%*	0%	6.3%	N/A	2.3 months^1^	5.1 months
BRAF/MEK inhibitors										
Dabrafenib + Trametinib	Davies et al. (2017)^75^ (COMBI-MB)	II	BRAF V600E, asymptomatic, no prior local brain therapy	76	0%	0%	58.0%	78.0%	6.5 months^4^	10.8 months
			BRAF V600E, asymptomatic, with prior local brain therapy	16	N/A	0%	56.0%	88.0%	7.3 months^4^	24.3 months
			BRAF V600D/K/R, asymptomatic, with/without prior local therapy	16	N/A	0%	44.0%	75.0%	8.3 months^4^	10.1 months
			BRAF V600D/E/K/R, symptomatic, with/without prior local therapy	17	N/A	0%	59.0%	82.0%	4.5 months^4^	11.5 months

^a^Radiation modality (WBRT vs SRS) is often not specified. SRS-only percentages are marked with asterisks (*).

^b^Prior TKI or ICI percentages listed relative to the study drug of interest (eg, prior ICI in an immunotherapy trial).

^c^Median unless otherwise stated.

Response duration endpoints: ^1^CNS PFS, ^2^PFS, ^3^DOR, ^4^CNS DOR, ^5^TTP, ^6^CNS TTP.

Abbreviations: N, number; BM, brain metastases; CNS, central nervous system; RT, radiation therapy; TKI, tyrosine kinase inhibitor; ICI, immune checkpoint inhibitor; ORR, objective response rate; DCR, disease control rate; NSCLC, non-small cell lung cancer; EGFR, epidermal growth factor receptor; ALK, anaplastic lymphocyte kinase; HER2, human epidermal growth factor receptor 2; PFS, progress-free survival; DOR, duration of response; TTP, time to progression; mOS, median overall survival; N/A, not applicable; NE, not evaluable; NR, not reached; WBRT, whole brain radiation therapy; SRS, stereotactic radiosurgery.

Many patients presenting with brain metastases will likely need both systemic therapy and local brain-directed therapy over their treatment course. Generally, patients who are symptomatic from higher burden of intracranial disease will require brain-directed therapy first. However, in patients with asymptomatic brain metastases, lower volume CNS disease, or extensive extracranial disease, systemic therapy prior to brain-directed therapy would be more appropriate. An active area of research is the timing of targeted therapies in relation to radiation therapy. For example, in select cases where patients have small asymptomatic brain metastases from melanoma or ALK rearrangement-positive NSCLC or EGFR-mutated NSCLC, it is reasonable to hold on treating with radiation to see if systemic therapy can control the brain metastases assuming close symptom and imaging surveillance. A recent study demonstrated EGFR-mutated NSCLC patients with CNS metastases have been shown to benefit from higher dosing of Osimertinib 160 mg PO daily for improved CNS control.^76^ However, a multicenter study of BM from EGFR-mutant NSCLC found that upfront use of TKI compared to SRS or WBRT was associated with significantly decreased overall survival.^77^ The optimal treatment of metastases with CNS-penetrant options remains one of the most important questions in contemporary neuro-oncology, with clinical trials ongoing, One such trial is the TROG-OUTRUN study (NCT03497767), a phase II study randomizing patients to Osemertinib alone to up-front SRS followed by Osemertinib therapy. Results are expected by early 2024.

Immune checkpoint inhibitors increase immune response to malignancy by blocking cytotoxic T-lymphocyte-associated antigen (CTLA-4) and programmed death 1 pathways.^78^ These agents, either as single or double agent, or in combination with chemotherapy and/or radiation, have demonstrated impressive CNS efficacy in specific solid tumor malignancies, primarily melanoma^79–82^ and NSCLC^83–85^ (Table 1). As with targeted agents, the disease control rate of most immune checkpoint inhibitor regimens is highest among patients with small, asymptomatic brain metastases. Furthermore, immunotherapeutics often trigger a strong inflammatory response in the CNS with resultant vasogenic edema and increased risk of radiation necrosis or treatment-related imaging changes, carrying with it a risk of increased symptom burden in patients with larger, symptomatic metastases.^86^ As such, up-front surgical resection or combination of radiation and immunotherapy is often employed in the interest of gaining better CNS control.^86,87^ There is currently a great deal of interest in the potential synergistic effects of immunotherapy combined with SRS for treatment of brain disease, with multiple trials ongoing.^88^

There is further mounting evidence that targeted agents and immunotherapies have a synergistic effect with radiation therapy across multiple tumor types.^88–90^ One study investigating immunotherapy administration for melanoma brain metastases found significant percent reduction in tumor volume when immunotherapy was administered within 4 weeks of SRS compared to treatment separated by more than 4 weeks.^91^ Another meta-analysis of 6 studies analyzing patients with HER-2 positive breast cancer brain metastases that received targeted agent lapatinib in addition to radiation therapy demonstrated increased local control.^92^ Clinical trials are urgently needed to study the timing with regards to combining SRS with targeted agents and immunotherapies.^89^

Future directions in the application of targeted therapy and immunotherapeutics rely on understanding tumor evolution, environment, and differences in molecular profile between the primary tumor and metastatic disease. Genetic divergence between brain metastases and the primary neoplasm may lend to decreased responses of CNS disease to targeted agents. In an analysis of 86 matched primary neoplasms and brain metastases, branched evolution resulted in 53% of brain metastases harboring clinically informative mutations not present in the primary malignancy.^93^ Importantly, these brain metastases, while frequently genomically distinct from the primary tumor, were similar to other brain metastases. Recent deep sequencing of lung cancer metastatic to the brain revealed metastases-specific genetic mutations that are targetable with small molecules.^94^ Therefore, confirmation of the genetic signature of brain metastases via surgical pathology or analysis of circulating tumor cells^95^ could provide important therapeutic data. Further work is needed to assess how frequently actionable mutations are discovered among brain metastases and whether candidate drugs can be safely added to existing systemic therapy without prohibitive toxicities. In the era of targeted therapies based on genetic mutations, biopsy for pathologic analysis or advancements in diagnosis via cell free DNA circulating in cerebrospinal fluid, are increasingly important to help guide management of these lesions.

## Management Algorithm

**Figure 1.**
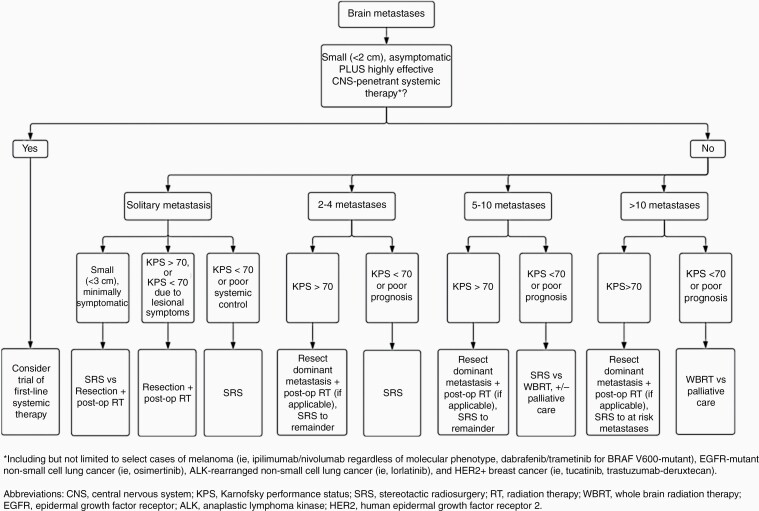
Treatment algorithm to guide clinical decision making for treatment of brain metastases.

Here we condense the data presented in this review into treatment algorithms to help guide clinical decision-making (Figure 1). The goal of these decision-making pathways is to simultaneously maximize oncologic efficacy and patient quality of life. The latter is achieved by favoring surgical resection when appropriate, employing targeted therapy when possible and minimizing radiation to symptomatic lesions or “at-risk” lesions. While the “at-risk” designation is inherently subjective, experienced practitioners intuitively understand lesions where current size, future growth, or edema may cause future neurologic sequalae. A multi-modal approach is often utilized in the treatment of metastases, combining resection, targeted therapy, immunotherapy, and/or radiation. Again, for patients with reasonable performance status and large, symptomatic lesions, surgery should be offered to relieve mass effect and reduce steroid dependence. Moreover, in our institutional experience, systemic therapies tend to not perform well in solid brain metastases over 2–3 cm. For small volume oligometastatic disease, we favor definitive treatment of all lesions given the current evidence and high efficacy of radiosurgical obliteration. On the other hand, for patients with a CNS-penetrant option and >5 asymptomatic lesions, it is reasonable to trial systemic therapy before offering radiosurgery, or proceed with SRS if technically feasible followed by systematic therapy, given support for advantages in both approaches.^77,91^ Regardless of approach, new data supports a short time interval between administration of radiation and targeted therapy or immunotherapy.

## Continued Evolution of WBRT

While SRS to multiple targets is increasingly utilized in treatment of brain metastases in tertiary cancer-care centers, this may not be feasible in all settings, especially in lower resource centers. In addition, in the case of disseminated leptomeningeal disease, WBRT remains the only radiotherapeutic option.^96^ A recent phase II trial even suggests that radiation of the entire neuroaxis with proton radiation provides superior progression-free survival (PFS) and overall survival compared to traditional photon-based radiation of only the diseased areas.^97,98^ An active area of investigation focuses on abrogating the negative effects of WBRT. Initial efforts focused on the use of neuroprotective drug memantine given at the time of WBRT. In a placebo-controlled trial, the use of memantine did not demonstrate a statistically significant effect on the primary neurocognitive endpoint of delayed recall at 24 weeks. One confounding factor was likely due to the rate of patient deaths during the trial.^99^ However, the drug does have a favorable side effect profile and did slow the rate of cognitive decline, making it a reasonable adjunct to patients receiving WBRT.

The most promising progress towards the goal of harm reduction is the use of HA-WBRT, which is based on protecting an adult neurogenic niche in the subgranular zone of the hippocampus.^7^ While this neurogenic niche is well-studied in rodents, its relevance to adult human biology remains controversial.^100^ Nevertheless, the strategy of HA-WBRT is bolstered by the observation that hippocampal dosimetry is correlated with cognitive decline in patients receiving radiation, specifically delayed^101^ and immediate word recall.^102^ A phase II trial of HA-WBRT^7^ enrolled 113 patients and 42 of those reached the 4-month endpoint. Furthermore, using a measurement of verbal learning/delayed recall, subjects exhibited a mean 7% decline from baseline, compared to 30% seen in historical controls. These promising results led to a phase III study which randomized 518 patients to receive WBRT with memantine versus HA-WBRT with memantine; overall risk of cognitive failure was significantly less (hazard ratio: 0.74) in the HA-WBRT group. At 6 months, patients had significantly less objective and subjective cognitive symptoms in the HA-WBRT group, with no effect on overall survival or PFS.^9^ This optimized HA-WBRT and memantine regimen is being compared to SRS for 5–15 metastases in an ongoing phase III clinical trial “A Phase III Trial of SRS Compared With Hippocampal-Avoidant WBRT (HA-WBRT) Plus Memantine for 5 or More Brain Metastases” ^52^ (Clinical Trial Identifier: NCT03550391). Another clinical trial “Phase III Trial of SRS Versus HA-WBRT for 10 or Fewer Brain Metastases From Small Cell Lung Cancer” is currently recruiting participants to determine if there is a difference in memory loss prevention and thinking ability between SRS and HA-WBRT.^103^

As discussed, the majority of patients with multiple brain metastases will die of systemic disease and not of their intracranial disease. It is on this basis that we favor SRS over WBRT in the treatment of multiple intracranial metastases with no upper limit. While SRS offers inferior distant control of metastases, this does not translate into a survival benefit. It is likely an individual will succumb to their systemic disease prior to having distant brain metastases failure contributing significantly to overall survival. While the generalizations above apply to the majority of individuals, there is still a subset of patients who will die of intracranial disease progression. For this population, a discussion of salvage radiation versus supportive care may be appropriate, and work is aimed at identifying which patients are at risk of suffering a neurological death.

For individuals who continue to progress through SRS, salvage WBRT is considered a treatment option. A retrospective study focused on the need for salvage radiotherapy after initial SRS found that just 27% of patients would require salvage therapy, and the average time of radiographic PFS before requiring retreatment was 6 months.^104^ In terms of choice of salvage therapy after local failure, salvage SRS has been found to be safe and effective, although associated with increased rates of radiation necrosis.^105^ The use of salvage SRS versus WBRT for local intracranial failure remains an open question. However recent data presented as an abstract suggests superior outcomes with SRS and a prospective trial to address this question is ongoing.^106^ Other options for the treatment of recurrent, previously irradiated metastases under investigation include open surgical resection with or without re-irradiation,^107^ Laser Interstitial Thermal Therapy (LITT)^108^ and repeat resection with brachytherapy using isotopes such as Cesium-131.^109^

## Future Directions and Unanswered Questions

### Timing and Sequencing of SRS Relative to Operative Resection

An area of active investigation is the timing and sequencing of delivering SRS relative to resection of brain metastasis. The importance of early treatment in the postoperative setting for local control was highlighted by studies finding the strongest predictor of local recurrence for brain metastases after postoperative SRS was the time to SRS.^110,111^ Patients who underwent SRS at 4 weeks or sooner from surgical resection had significantly increased local recurrence-free survival when compared to patients who underwent SRS more than 4 weeks after surgical resection.^111^ For patients who received SRS greater than 8 weeks after surgery, there was no significant difference in local recurrence rates compared to patients who never received postoperative SRS.^110^ Recently, the concept of pre-operative SRS has been explored with promising results.^112^ A major benefit of pre-operative SRS is there is no longer a delay in delivering radiation after surgery, which can vary greatly especially depending on a patients clinical course.^113^ In addition, both irregularity of the postoperative bed and seeding of cerebrospinal fluid with malignant cells at time of surgical resection are of less concern in pre-operative SRS. In a study of 47 patients treated with neoadjuvant SRS prior to resection of brain metastases, Asher et al. reported a local control rate of 97.8% at 6 months and 85.6% at 12 months.^114^ In a study of 180 patients, of whom 36.7% underwent pre-operative SRS, there was no significant difference in rates of overall survival, local or distal brain recurrence between pre- and postoperative SRS, but there was significant lower rates of leptomeningeal disease and symptomatic radiation necrosis is the cohort who received preoperative SRS.^115^ Currently, there are 4 ongoing clinical trials studying pre-operative SRS for brain metastases.^112,116–119^ Of note, there is no consensus in the postoperative setting about whether single-fraction or 3-fraction SRS provides the best local control.

### Other Therapeutic Alternatives: Brachytherapy, LITT, and Optune-Tumor Treating Fields

The use of brachytherapy seeds in the resection cavity provides an alternative method of post-resection radiation to external radiation.^42,120^ As mentioned above, there is limited time window that postoperative SRS is maximally effective in preventing local recurrence. If there are anticipated difficulties with scheduling a patient for postoperative SRS, brachytherapy seeds are a safe and effective alternative. In a Phase I/II study of resection and intraoperative cesium-131 radioisotope brachytherapy seed placement in 24 individuals, there were no cases of local recurrence or radiation necrosis with a median follow-up time of 19.3 months.^121^ In a matched pair analysis of patients treated with SRS versus cesium-131 radioisotope brachytherapy seeds after gross total resection of a brain metastasis, the local recurrence rate was significant lower in the brachytherapy seed cohort compared to SRS.^122^

LITT employs thermal ablation through a laser probe to target metastatic disease. For brain metastases, LITT has been utilized in the treatment of radiation necrosis, as well as brain metastases that have previously failed SRS.^123,124^ Another novel method of treatment currently being studied in a phase II single-arm clinical trial is the use of Optune-Tumor Treating Fields for SCLC brain metastases. Optune device, a portable battery powered device, delivers continuous alternating electric fields to the brain through a skull cap.^118^

### KPS

The use of KPS to help guide eligibility for therapy should distinguish between low KPS due to systemic disease burden vs neurological deterioration in the setting of brain tumor that is more likely to reverse when the lesion(s) are treated. For example, in the landmark prospective observation study investigating the efficacy of SRS for the treatment of 5–10 brain metastases compared with 2–4, individuals with KPS < 70 with intracranial disease was a significant contributor to performance status were included in the study.^44^ Careful consideration should be given before utilizing KPS to determine eligibility for treatment.

### Brain Metastasis Velocity

The recently described Brain Metastasis Velocity (BMV) is a metric that attempts to quantify the risk of intracranial progression and neurologic death.^125^ The BMV score is simply calculated by dividing the number of new metastases since initial SRS treatment by the time since treatment, giving a measurement of metastases/year at the time of treatment failure. In the initial study, 737 patients were stratified to a low (BMV < 4), intermediate (4–13), and high-risk groups (>13), and these risk groups correlated with higher incidence of need for salvage WBRT and neurologic death. Subsequent studies found that the BMV score and its prognostic value was valid for predicting survival after multiple rounds of SRS (up to 4 treatments).^126^ BMV is now being increasingly utilized to triage patients to receive either salvage SRS or WBRT and BMV is being used also as a stratification variable in upcoming trials.^127^

## Limitations

Although we intend this review and generated algorithm to be a comprehensive summation of the literature to help guide treatment of brain metastases, consideration of unique patient factors must be taken into account by the treating physician. With improving systemic therapies and evidence to support a synergistic effect between SRS and such therapies, a discussion with the primary oncologist should be had on the appropriate use of new targeted molecules and immunotherapies concurrently or staged with radiation depending on the primary tumor type. We focus on the new diagnosis of brain metastases, and do not focus on the treatment of recurrence, progression, or pseudo-progression. The treatment of recurrent disease, including the use of salvage radiation versus other modalities, is an area of active investigation.

The treatment of high-risk surgical tumors likely to leave a postoperative deficit, require careful consideration regarding the goals of surgery and expected quality of life postoperatively. A surgery for palliation of symptoms is not effective if the patient develops a new deficit. Ultimately, the risk and benefit analysis of surgical resection is a multidisciplinary conversation between the surgeon, primary oncologist, and patient.

Finally, our algorithm presupposes a tertiary or quaternary health system in which there is access to neurosurgery, radiosurgery, targeted therapies, and contemporary multidisciplinary brain metastases care. SRS for >4 metastases is done routinely in academic centers but less routinely in the community setting. It is our hope that in the coming years, emerging clinical trials and increasing experience will lead to more widespread adoption of these techniques.

## Conclusion

Considering the expected increase in incidence and prevalence of brain metastases due to the overall enhanced survival of patients with systemic cancer in the era of immunotherapy and targeted chemotherapy, a clear algorithm for the surgical and radiosurgical management of this disease is needed. The management of solitary metastases is straightforward, with a plethora of good evidence: large or symptomatic tumors should be resected followed by SRS to the resection cavity for enhanced local control. We recommend surgical resection in any patient with a favorable performance status, or a patient with a poor performance status due to symptoms attributable to a dominant metastasis. Surgical resection provides palliation of symptoms and intracranial control for masses that otherwise cannot be treated with SRS due to size. No large body of evidence exists regarding the number of repeat resections considered reasonable or the number of individual metastases that should be resected. With regard to radiosurgery, the vast majority of studies to date suggest SRS is effective and safe for 1–4 metastases and should be favored over WBRT. Currently, there are no published randomized trials that state WBRT can be withheld for 5 or more metastases. However, the combination of large prospective and retrospective series examining the use of SRS monotherapy suggest that WBRT can be omitted upfront without impacting overall survival and have the benefit of avoiding the cognitive side effects of WBRT.

Effective brain metastasis control will be achieved with a combination of surgical resection, radiosurgery, and effective targeted and immune-modulating therapies. While much is settled regarding the treatment of oligometastatic disease, the treatment of multiple brain metastases continues to be an evolving balancing act between effective intracranial control, management of systemic progression, and avoidance of cognitive toxicity in a patient population that is experiencing longer overall survival. Understanding nuances of management algorithms based on studies of brain metastases from various primary tumor types will be increasingly important going forward. Further refinements to WBRT are being investigated, such as HA-WBRT, to decrease the cognitive side effects. Undoubtedly the addition of immunotherapy and targeted therapy, found to have synergistic effects with radiation, will shape practice in the coming years. Our proposed algorithm incorporates the best evidence and saves WBRT as a last-line salvage therapy.
